# Effect of salted water temperature on dynamic process and collapse behavior of the cavitation bubble

**DOI:** 10.1016/j.ultsonch.2025.107495

**Published:** 2025-08-05

**Authors:** Guihua Fu, Jing Luo, Weilin Xu, Jiguo Tang, Hang Wang

**Affiliations:** State Key Laboratory of Hydraulics and Mountain River Engineering, Sichuan University, Chengdu 610065, China

**Keywords:** Shock wave, Microjet, Secondary cavitation, Bubble collapse, Salted water temperature

## Abstract

How to efficiently utilize cavitation to promote chemical reactions or degrade microorganisms is a concern in the fields of biological and chemical engineering. Changes in liquid temperature can affect bubble dynamics, thereby influencing chemical reaction rates or microbial degradation efficiency. This study used the method of corona discharge induced bubbles, combined with high-speed camera system and temperature control system, to investigate the effect of salted water temperature on the bubble collapse behavior in the free field and below the liquid surface. In the free field, it was found that salted water temperature affected the maximum radius, minimum first contraction radius, expansion time and collapse time of bubble. And the above parameters showed an increasing trend with the increase of salted water temperature. Further measurements using high-frequency pressure testing system revealed that as salted water temperature increased, the pressure peak of shock wave and relative energy gradually decreased. The above experimental results were mainly due to the effect of salted water temperature on evaporation and condensation rate, which led to different microjet and shock wave behaviors under varying boundary conditions. The study found that as salted water temperature increased (10 ℃ − 40 ℃), the microjet velocity and the maximum pressure peak of the shock wave below the free liquid surface gradually decreased. A novel phenomenon was observed where bubble expansion in high-temperature salted water induced secondary cavitation, forming a “satellite bubble” pattern. These findings provided a new idea to controlling cavitation intensity through temperature in the actual cavitation scene.

## Highlights

• Increase in salted water temperature can delay the bubble collapse process.
• Increase in salted water temperature can attenuate the intensity of the shock wave.
• Increase in salted water temperature can reduce the microjet velocity.
• Secondary cavitation occurs during bubble expansion in high-temperature salted water.

## Nomenclature

**Symbols**: **Symbol Meaning (Unit)**
*R*: The equivalent radius of the bubble (mm)
*R*_max_: The maximum radius of the bubble in the first cycle (mm)
*R*_r-max_: The maximum radius of the bubble in the second cycle (mm)
*R*_min_: The minimum radius of the bubble in the first cycle (mm)
*R*_s_: The radius of the secondary cavitation bubble (mm)
*L*: The distance between the center of the bubble and the sensor measuring point (mm)
*L*_1_: The distance between the center of the secondary cavitation bubble and the center of the bubble (mm)
*γ*_1_: The relative distance between the center of the bubble and the sensor measuring point
*γ_2_*: The relative distance between the center of the bubble and the free salted water surface
*γ_3_*: The relative distance between the center of the bubble and the center of the secondary cavitation bubble
*γ_4_*: The dimensionless ratio of the radius of the secondary cavitation bubble to the maximum radius of the bubble in the first cycle
*T*: The temperature of salted water (℃)
*t*: The evolution time of the bubble (ms)
t*: The first evolution period of the bubble (ms)
*t_c_*: The evolution time of the bubble shrinking from the maximum radius to the minimum radius in the first cycle (ms)
*t*_e_: The evolution time of the bubble from generation to maximum volume in the first cycle (ms)
*t*_j_: The formation time of the bubble-induced microjet
*t*_j_*: The evolution time of the bubble-induced microjet
*V*: The maximum velocity of the bubble-induced microjet (m/s)
*ρ*: The density of salted water (kg/m^3^)
*P*: The impact pressure of the shock wave generated by the bubble collapse (MPa)
*P*_max_: The pressure peak value of the collapse shock wave (MPa)
*P*_max_*: The maximum pressure peak value of the shock wave below the salted water surface (MPa)
*E*_s_: The shock wave energy generated by the bubble collapse (J)
*E*_max_: The maximal mechanical energy of the bubble (J)
*E*_r-max_: The mechanical energy of the rebound bubble (J)
*E*_s_*: The shock wave energy generated by the bubble collapse below the salted water surface (J)
*E*_max_*: The maximal mechanical energy of the bubble below the salted water surface (J)

## 1 Introduction

Cavitation phenomenon is widely present in the field of biological and chemical engineering, such as the use of ultrasound generated bubbles to accelerate chemical reactions [1,2], and hydrodynamic cavitation can degrade microorganisms in wastewater on a large scale [3,4]. However, in the cavitation scenario in the field of biological and chemical engineering, the liquid temperature would vary with different application scenarios [5,6], and some may even differ greatly [7]. This difference in the dynamic characteristics of the cavitation bubble at different temperatures would in turn affect the utilization efficiency of cavitation at the macro level.

The liquid temperature can affect its physical properties such as saturation vapor pressure, vaporization rate, surface tension, and viscosity. Florschuetz and Chao [8] conducted a theoretical analysis of the bubble under spherically symmetric conditions in water and ethyl alcohol. They proposed three types of bubble collapse scenarios: liquid inertia control, heat transfer control, and two important intermediate scenarios. Liu et al. [9] analyzed the evolution of the bubble in water at different temperatures using numerical simulation and experimental methods. They found that as the water temperature increased, the maximum radius and evolution period of the bubble increased. Dular and Coutier Delgosha [10] first measured the temperature field of the surrounding distilled water during single bubble evolution. They found that the experimental results were basically consistent with the temperature changes predicted by the “thermal delay” model (Brennen [11]). Qin and Alehossein [12] studied the temperature variations and heat transfer during bubble collapse in water by combining the Rayleigh Plesset equation with CFD modeling. Zhang et al. [13] studied the evolution of laser-induced bubble in distilled water at different temperatures under the same laser energy condition. They found that as the temperature of the solution increased, the influence of the phase change rate at the bubble interface on the maximum radius, the first minimum radius, and the oscillation period of the bubble gradually strengthened. Through the study of five phase change models, Liu et al. [14] investigated five phase-change models and found that the temperature difference between the bubble interface and the saturation temperature in the liquid affects the accuracy of simulating bubble condensation. Through experimental and numerical simulation methods, Phan et al. [15] found that the maximum radius, first minimum radius, and collapse time of the bubble all increase with the increase of water temperature. Zhan et al. [16] proposed a theoretical model of cavitation bubble dynamics and found that phase transition was an important cause of energy loss in laser-induced and spark-induced cavitation bubble in water, and this energy loss gradually increased with the increase of Mach number and initial vapor proportion in the bubble. Pei et al. [17] studied the dynamic characteristics of spark-induced cavitation bubble in water at temperatures ranging from 23 °C to 90 °C and found that when the temperature exceeds 70 °C, secondary cavitation occurs near the surface of the bubble when it expands to its maximum volume.

Since the discovery of cavitation phenomena, Rayleigh [18] first proposed the equation of motion for a single spherical bubble in an unbounded domain of liquid. In addition to theoretical research, many scientists have also observed and analyzed the evolution [[19], [20], [21]] and collapse characteristics [[22], [23], [24]] of the bubble. Blake and Gibson [25] studied the microjet generated during the evolution of the bubble near the rigid boundary and free liquid surface. Wang et al. [26] studied the interaction between the bubble and the free liquid surface through numerical simulation. They found three different collapse modes of the bubble when the distance between the free liquid surface and the center of the bubble was studied from 1.5 times the maximum radius of the bubble. Pecha and Gompf [27] used a stripe camera to record the evolution of cavitation bubbles in water, revealing that the propagation speed of the shock wave reached approximately 4000 m/s. Akhatov et al. [28] proposed a mathematical model for the spherical motion of a laser bubble in distilled water, considering compressibility, heat and mass transfer, and evaporation–condensation at the bubble interface. They found that the calculated results were in good agreement with the evolution of the bubble radius and shock wave intensity measured by experiments. Supponen et al. [29] studied shock waves generated by nonspherical laser bubbles in deionized water at varying distances from a free water surface. Based on experimental and numerical simulation methods, Wang et al. [30] studied the dynamic characteristics of the interaction between laser-induced bubble and a spherical particle in deionized water. Using a combination of experiments, numerical simulations, and theoretical analysis, Zhang et al. [31] investigated how physical factors such as gravity and inertia affect the interaction between a cavitation vortex ring and the free water surface.

From the above literature review, it could be seen that temperature changes in liquids could have an impact on the evolution characteristics of the bubble. However, few studies had explored the effect of liquid temperature on the dynamic process of the bubble collapse, microjet velocity, and shock wave intensity under different boundary conditions. Therefore, this article used single-electrode high-voltage corona discharge to induce cavitation bubbles, combined with a high-speed photography system and a high-frequency pressure testing system to investigate the dynamic characteristics of the bubble in the range of 10 ℃ − 90 ℃. Based on the clear influence of salted water temperature increase on the bubble evolution, this study had innovatively discovered the influence of salted water temperature increase on the shockwave energy during the bubble collapse. Furthermore, the effect of the increase in salted water temperature on the microjet and shock wave evolution during the bubble collapse below the free surface was studied, using the surface to induce asymmetric collapse. The research in this article filled the gap in understanding the influence of salted water temperature on the dynamic characteristics of the bubble.

## 2 Experimental methods and repeatability

### 2.1 Experimental methods

**Fig. 1:**
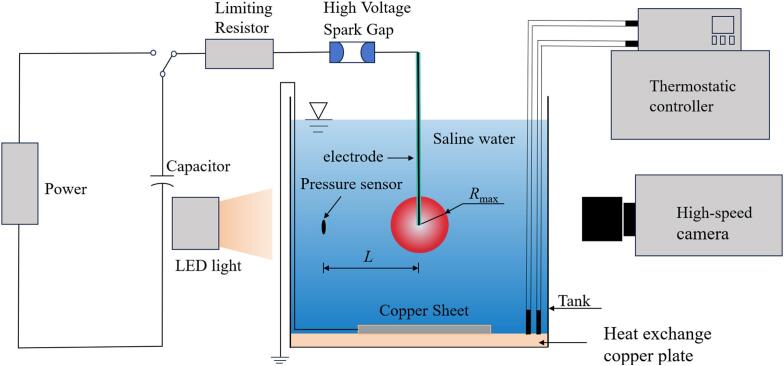
Experimental device diagram.

In order to study the evolution and collapse characteristics of the bubble in salted water at different temperatures, this study utilized the corona discharge-induced cavitation bubbles system, high-speed photography system, parallel light system, low-temperature thermostatic system, and transient pressure measurement system to observe and collect data on the bubble, as shown in Fig. 1. The ambient atmospheric pressure during the experiment was 96 kPa.

The corona discharge-induced bubble system consisted of a charging circuit and a discharging circuit. An underwater stainless steel electrode with a radius of 0.3 mm wrapped in insulation was connected to a discharge circuit through a high-voltage spark gap. The endpoint of the stainless steel electrode was in contact with salted water. At the same time, a grounding copper plate was placed at the bottom of the water tank. Water contained a certain salinity, and free particles in salted water could act as charges. When discharging underwater electrodes, the current at the electrode tip is extremely large, resulting in the formation of the bubble [32]. In each experimental condition, this study ensured that the voltage and resistance in the charging circuit were the same, and the ball gap spacing in the discharging circuit was the same. Each experimental condition was repeated 8 times. Consider temperature control of salted water and reflection of shock waves. This study selected a transparent glass tank with dimensions of 28 cm × 28 cm × 30 cm. The endpoint of the electrode was close to the center of the water tank, and the depth of salted water in the tank was 27 cm. In order to eliminate the influence of the salted water surface and the inner wall of the glass tank on the bubble, the distance between the electrode tip and both the free surface and the tank walls was maintained at more than 10 times the maximum bubble radius for all test conditions [33].

The evolution time of shock wave and microjet generated by the bubble collapse was very short. In order to capture the evolution details of the bubble in salted water at different temperatures as much as possible, this study utilized a high-speed photography system to observe bubble microjet and collapse shock wave. The maximum shooting speed of the high-speed camera (Fastcam SA-Z, Photron Inc, Japan) was 1,000,000 fps (Frame per second). Due to the millimeter scale of the bubble in this experiment, the evolution of the bubble microjet below the salted water surface was captured using a macro lens (NIKKOR 105 mm) during the experiment. The macro lens used under other experimental conditions was NIKKOR 85 mm. In order to balance the shooting speed and image clarity of high-speed cameras, the shooting resolution selected in the experiment was 256 pixel × 256 pixel. When capturing the evolution of the bubble in a free field, the shock wave and microjet below the salted water surface, the actual dimensions of a single pixel were 0.192 mm/pixel, 0.244 mm/pixel, and 0.125 mm/pixel, respectively. In order to ensure a clear shot of the shock wave front, the exposure time was selected to be 0.25 µs.

The shock wave propagation speed in the bubble evolution is higher than the speed of sound in water in the proximal region [23]. Due to the higher density of salted water at the wavefront compared to the surrounding medium during propagation, a distinct wavefront is visible in the high-speed camera images. Schlieren photography was employed to capture the shock wave propagation in experiments of the shock wave [34]. For investigating bubble dynamics and microjet formation, a 200 W LED light source was utilized as supplemental illumination.

Precise temperature control of the salted water in the glass tank was critical. To better control the salted water temperature, a low-temperature thermostatic system (DC-0520, Nuoda Instrument Technology Co. Ltd) was used in this study. The tank volume of the instrument is 20 L, the temperature control range is −5 ℃-100 ℃, the temperature display accuracy is 0.1 ℃, and the sensor is a platinum resistance PT100. During the experiment, the temperature of the deionized water in the tank of the low-temperature thermostatic system was heated or cooled to the required experimental temperature. Subsequently, the outlet and inlet of the low-temperature thermostatic system were connected to the heat exchange copper plate, respectively. Finally, the water circulation switch of the low-temperature thermostatic system was turned on to facilitate heat exchange with the salted water in the glass tank. as shown in Fig. 1. In addition, this study used mercury thermometers to measure the salted water temperature.

The impact pressure of collapse shock wave is very high. A piezoresistive pressure sensor (Test Electronics Information Co. Ltd) was used to measure shock waves of the bubble in this study. The maximum measuring range of the sensor is 20 MPa, the rise time is 2 µs, the response frequency is 0 kHz − 500 kHz, and the temperature range is 0 ℃ − 80 ℃ [35]. According to the calibration of the voltage and pressure for the sensor, within the temperature range of 0 ℃ − 80 ℃, the influence of temperature on the sensitivity of the sensor is negligible. When measuring shock waves, this experiment chose to measure the impact pressure at a distance of three times the maximum radius of the bubble from the center of the bubble. When studying the shock wave and microjet of the bubble below the salted water surface, this research chose a distance 1.2 times the maximum radius of the bubble from the bottom of the salted water surface as the center of the bubble.

**Fig. 2:**
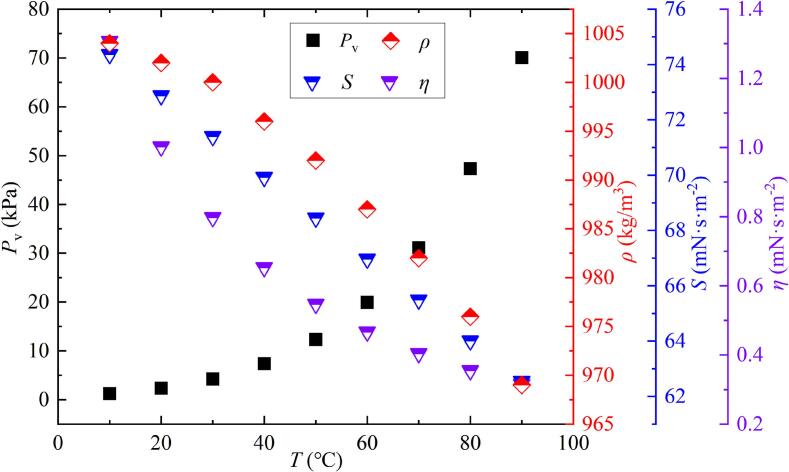
Physical parameters of liquids at different temperatures.

To better study the effect of salted water temperature on bubble evolution and collapse characteristics, the experiment used a mixture of salt and deionized water. The salinity was set at 6.8 ‰. The salted water temperatures for the experiment were 10 ℃, 20 ℃, 30 ℃, 40 ℃, 50 ℃, 60 ℃, 70 ℃, 80 ℃, and 90 ℃, respectively. The changes in saturated vapor pressure *P*_v_ [36], viscosity *η* [36], and surface tension *S* [36] of deionized water with increasing temperature of deionized water were shown in Fig. 2. The change in density *ρ* of salted water with increasing salted water temperature was shown in Fig. 2. Previous studies have shown that the variations of the above parameters in seawater with increasing temperature were essentially consistent with those in deionized water [36,[37], [38], [39], [40]]. Therefore, this study used the variations of physical parameters in deionized water as temperature increased to reflect the variations of physical parameters in salted water as temperature increased.

### 2.2 Corona discharge induced cavitation bubbles repeatability

**Fig. 3:**
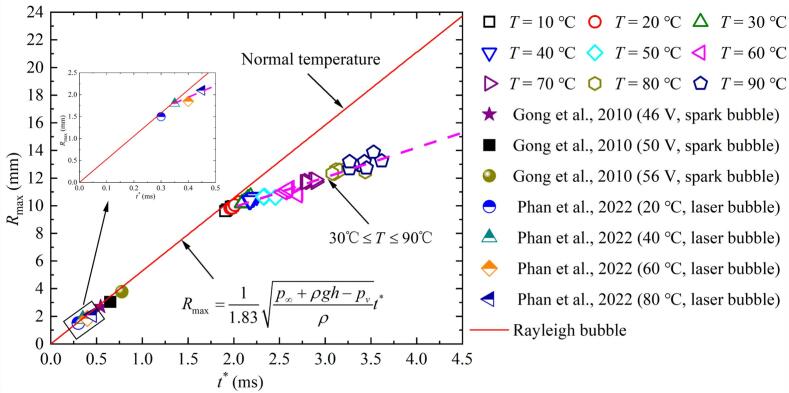
Repeatability verification of corona discharge-induced cavitation bubbles experiments.

In order to verify the experimental repeatability of Corona discharge induced cavitation bubbles, this study repeatedly observed the relationship between the maximum radius *R*_max_ and the first period t* of the bubble in salted water at the same temperature, and compared it with the relationship between *R*_max_ and t* in Phan et al. [15] and Gong et al. [24] and the relationship between *R*_max_ and t* in Rayleigh bubble, as shown in Fig. 3. The relationship between *R*_max_ and t* of Rayleigh bubble in unbounded domains can be calculated using the following formula [18]:

(1) $$t^{*}=1.83\sqrt{\frac{\rho }{p_{\infty }+\rho gh-p_{v}}}R_{\max}$$

In the formula, the salted water density *ρ* with a salinity of 6.8 ‰ at 20 ℃, taken as 1002 kg/m^3^; the saturated vapor pressure $p_{v}$ of the bubble is taken as 2.34 kPa; $p_{\infty }$ is the environmental pressure, taken as 96 kPa; g is the gravitational acceleration, taken as 9.8 m/s^2^; h is the salted water depth between the salted water surface and the center of the bubble, taken as 0.12 m.

As can be seen from Fig. 3, under salted water temperature conditions of 10 °C and 20 °C, the relationship between *R*_max_ and t* of corona-induced bubble was consistent with the relationship between *R*_max_ and t* of spark bubble [24] and Rayleigh bubble. The variation of *R*_max_ and t* with increasing salted water temperature observed in this study for liquids between 30°C − 90°C was generally consistent with the results of Phan et al. [15], roughly following the trend indicated by the magenta dashed line. The difference was that the bubble scale in this study was larger, leading to more pronounced deviation effects, whereas the laser-induced bubble in Phan et al. [15] was smaller in size. In addition, as shown in Fig. 3, under the same conditions, the differences in the *R*_max_ and t* in the corona discharge-induced cavitation bubbles experiment were small, indicating good repeatability of this experimental method.

## 3 Results and analysis

### 3.1 Effect of salted water temperature on bubble evolution and collapse in a free field

To quantitatively investigate the evolution and collapse characteristics of single bubble in a free field under varying salted water temperature conditions, this section would analyze three key aspects: the evolution form, collapse characteristics, and energy distribution of the bubble.

#### 3.1.1 Effect of salted water temperature on the bubble evolution and collapse

**Fig. 4:**
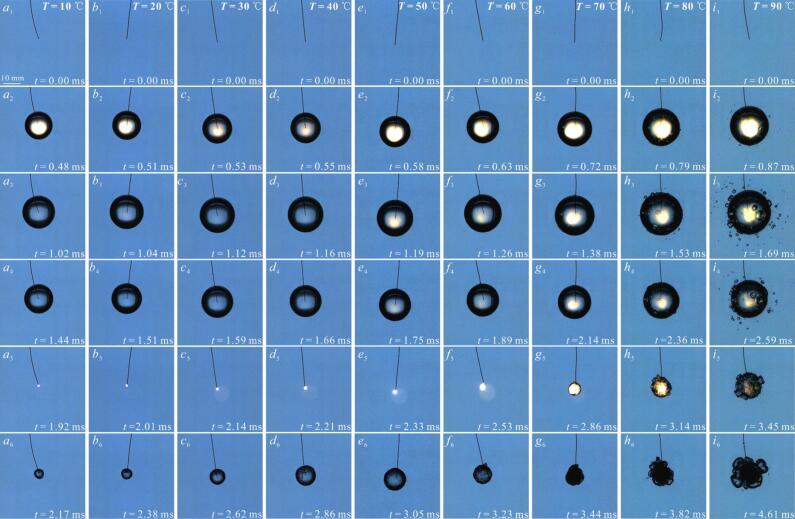
High-speed photographic images of the effect of salted water temperature on the bubble evolution in a free field. The subscripts 1, 2, 3, 4, 5, and 6 for each operating condition represented the evolution process of the bubble. (shooting rate: 180,000 fps; exposure time: 0.35 µs; (*a*) *T* = 10 ℃; (*b*) *T* = 20 ℃; (*c*) *T* = 30 ℃; (*d*) *T* = 40 ℃; (*e*) *T* = 50 ℃; (*f*) *T* = 60 ℃; (*g*) *T* = 70 ℃; (*h*) *T* = 80 ℃; (*i*) *T* = 90 ℃).

Fig. 4 showed high-speed photographic images of the bubble evolution in a free field as the salted water temperature increased from 10 °C to 90 °C.

In Fig. 4 (*a*-*i*), the salted water temperature *T* increased from 10 ℃ to 90 ℃. In Fig. 4 (*a_3_*-*i_3_*), the evolution time *t* for the first expansion to maximum volume of bubble increased from 1.02 ± 0.01 ms to 1.71 ± 0.08 ms, with the corresponding maximum radius *R*_max_ increasing from 9.80 ± 0.13 mm to 13.09 ± 0.35 mm. When *T* ranged from 10 °C to 60 °C, the bubble maintained relatively good sphericity during expansion to maximum volume. When *T* ranged from 70 °C to 90 °C, secondary cavitation bubbles were generated on the surface of the bubble and in the nearby salted water when the bubble expanded to its maximum volume. This phenomenon would be discussed in detail in Part 3.3 of this study. In Fig. 4 (*a_5_*-*i_5_*), the evolution time *t* for the first contraction to minimum volume of the bubble increased from 1.95 ± 0.03 ms to 3.48 ± 0.24 ms, with the corresponding minimum radius *R*_min_ increasing from 0.79 ± 0.03 mm to 8.08 ± 0.27 mm. When *T* ranged from 10 °C to 50 °C, the bubble generally collapsed in a point like manner. This collapse form was basically consistent with the research results of Supponen et al. [29] under normal temperature conditions. When *T* ranged from 60 °C to 90 °C, both the surface irregularity during bubble contraction to minimum volume and the corresponding minimum radius exhibited a gradual increase with increasing salted water temperature.

When the bubble contracted to its minimum volume for the first time, it would rebound [41]. In Fig. 4 (*a_6_*-*i_6_*), the evolution time *t* for the first rebound to maximum volume increased from 2.20 ± 0.02 ms to 4.67 ± 0.24 ms, with the corresponding rebound maximum radius *R*_r-max_ increasing from 3.06 ± 0.16 mm to 9.72 ± 0.56 mm. The difference was that the maximum radius of the rebounding bubble at 50 ℃ was higher than that at 60 ℃ − 80 ℃. The irregularity of the maximum volume of the rebound bubble gradually increased with increasing salted water temperature.

Through high-speed photographic observations of single bubble evolution under varying salted water temperatures in Fig. 4, it could be observed that as salted water temperature increased, the maximum radius *R*_max_, first evolution period t*, and minimum radius *R*_min_ of the bubble during first collapse all showed increase. Meanwhile, the expansion time *t*_e_, collapse time *t*_c_ and maximum rebound radius *R*_r-max_ of the bubble also changed. This reflected the effect of the increase in salted water temperature on the evolution and collapse characteristics of the bubble to some extent. Therefore, through repetitive experiments on bubbles under different salted water temperature conditions, this study divided the salted water temperature into three zones: the inertial effect zone (10°C − 30°C), the transition zone (30°C − 60°C), and the heat transfer zone (60°C − 90°C).

**Fig. 5:**
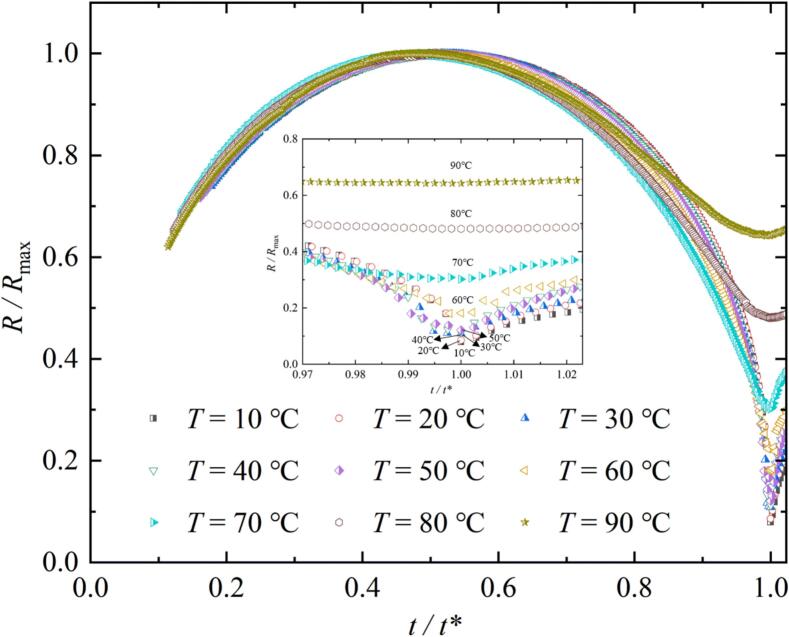
Variation of the ratio of the radius *R* to the maximum radius *R*_max_ of the bubble as the ratio of the evolution time *t* to the first evolution period t* of the bubble under different salted water temperature conditions.

Fig. 5 showed the variation of the ratio of the radius *R* of the bubble to its maximum radius *R*_max_ under different temperature conditions as the ratio of the evolution time *t* to the first evolution period t* of the bubble. *R* represented the equivalent radius during the bubble evolution process under different temperature conditions. In Fig. 5, when (t / t*) was equal to 1, the ratio of (*R / R*_max_) increased as *T* increased. The subplot in Fig. 5 was enlarged view of the minimum value of (*R / R*_max_) under different temperature conditions.

**Fig. 6:**
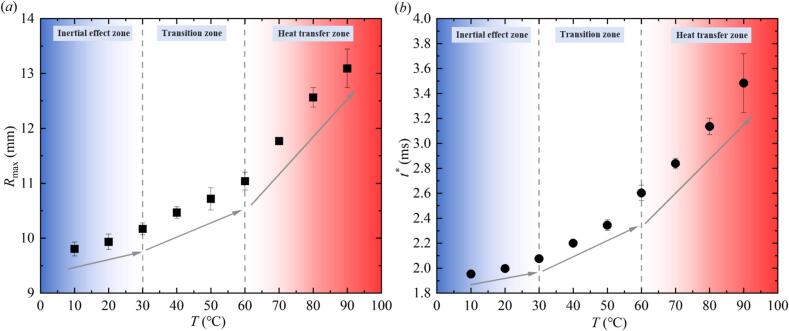
Effect of salted water temperature on the maximum radius *R*_max_ and the first evolution period t* of the bubble ((*a*) Effect of salted water temperature on the maximum radius *R*_max_ of the bubble; (*b*) Effect of salted water temperature on the first evolution period t* of the bubble).

Fig. 6 showed the variation of the maximum radius *R*_max_ and the first evolution period t* of the bubble under different salted water temperature conditions. In Fig. 6 (*a*), the *R*_max_ in the inertial effect zone, transition zone, and heat transfer zone gradually increased with the increased of *T*, and the magnitude of the increase (indicated by the gray arrow) was gradually increasing. The study that *R*_max_ increased with the increase of *T* was consistent with the research of Liu et al. [9] and Phan et al. [15]. The difference was that the experimental temperature range of this study was larger. In addition, in Fig. 6 (*b*), the t* in the inertial effect zone, transition zone, and heat transfer zone gradually increased with the increase of *T*, and the magnitude of its increase was also increasing.

**Fig. 7:**
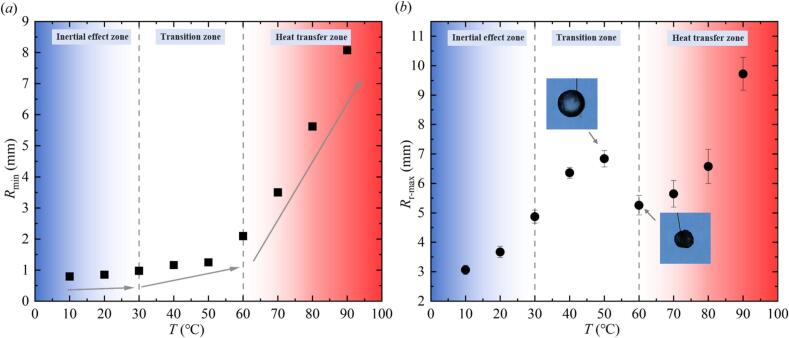
Effect of salted water temperature on the first collapse minimum radius *R*_min_ and the first rebound maximum radius *R*_r-max_ of the bubble ((*a*) Effect of salted water temperature on the first collapse minimum radius *R*_min_; (*b*) Effect of salted water temperature on the first rebound maximum radius *R*_r-max_, with photographic images of the bubble morphology at maximum rebound under 50°C and 60°C).

Fig. 7 further showed the variation of the first collapse minimum radius *R*_min_ and the first rebound maximum radius *R*_r-max_ of the bubble under different temperature conditions. In Fig. 7 (*a*), the *R*_min_ in the inertial effect zone, transition zone, and heat transfer zone gradually increased with the increase of *T*, and the magnitude of the increase also increased. In Fig. 7 (*b*), *R*_r-max_ in the inertial effect zone, transition zone and heat transfer zone also increased with the increase of *T*. The difference was that the average value *R*_r-max_ at 50 ℃ was higher than that at 60 ℃ − 80 ℃.

When *T* was 60 ℃, there may be two reasons for the sudden change in the maximum rebound radius of the bubble: on the one hand, the luminescence phenomenon during the contraction process of the bubble in salted water at 60 ℃ was more significant than that at 50 ℃, and part of the energy of the bubble was converted into light energy and emitted out. On the other hand, the bubble in salted water at 60 ℃ exhibited a higher degree of asymmetry during rebound than that at 50 ℃. Consequently, the maximum rebound radius of the bubble at 60 ℃ is smaller than that at 50 ℃.

**Fig. 8:**
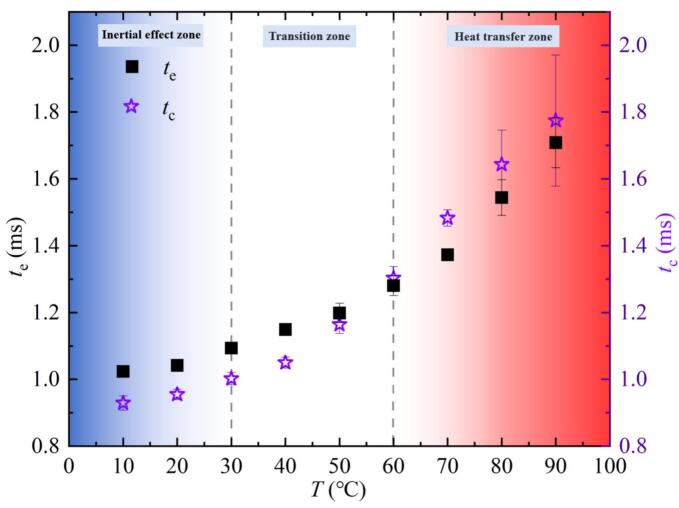
Effect of salted water temperature on expansion time *t*_e_ and collapse time *t*_c_ of the bubble in the first cycle.

The above contents analyzed the influence of salted water temperature variations on bubble evolution and collapse characteristics from a spatial perspective. Therefore, Fig. 8 showed the variation of expansion time *t*_e_ and collapse time *t*_c_ in the first cycle of the bubble under different temperature conditions from a temporal perspective. In Fig. 8, *t*_e_ and *t*_c_ gradually increased with the increase of *T*. Differently, as *T* increased, the relative magnitude of *t*_e_ and *t*_c_ varies. *t*_e_ in the inertial effect zone was greater than *t*_c_. The analysis results were consistent with the study by Qu et al. [42] at normal temperature. In the transition zone, when *T* was lower than or equal to 50 ℃, *t*_e_ was greater than *t*_c_. When *T* was 60 ℃, *t*_e_ may be less than or equal to *t*_c_. *t*_e_ was less than *t*_c_ in the heat transfer zone. Therefore, within the experimental scope of this study, 60 ℃ was roughly the critical temperature at which *t*_e_ and *t*_c_ were equal.

There may be two reasons for the occurrence of the critical temperature (60 ℃): on the one hand, during the bubble expansion stage, the vaporization rate at the bubble surface in high-temperature salted water was higher than the condensation rate, and also higher than the vaporization rate in low-temperature salted water. This led to *t*_e_ > *t*_c_ in low-temperature salted water and *t*_e_ < *t*_c_ in high-temperature salted water. On the other hand, during the bubble collapse stage, the condensation rate in high-temperature salted water was lower than that in low-temperature salted water [15]. This led to *t*_c_ < *t*_e_ in low-temperature salted water and *t*_c_ > *t*_e_ in high-temperature salted water.

From the images and data results of the above experiment, it could be found that the increase in salted water temperature had a significant impact on the evolution and collapse characteristics of the bubble. The fundamental reasons for the above research results were as follows: on the one hand, as salted water temperature increased, the saturated vapor pressure [17,36] and vaporization rate of salted water progressively increased. This led to the increase in the maximum radius, evolution period, expansion time of the bubble. This phenomenon was also observed in the literature [17]. On the other hand, when the salted water temperature increased, its condensation rate [15] gradually decreased, resulting in a gradual increase in the minimum radius of the bubble. Additionally, in our experiments, the influence of temperature changes on surface tension and viscosity resulted in a relatively small variation in bubble dynamic characteristics [9,43,44]. The effect of temperature on bubble dynamics was more significant than that of surface tension and viscosity, making it the primary factor. The differences in vaporization and condensation rates, surface tension, and viscosity at different salted water temperatures caused variations in bubble evolution and collapse, which in turn affected the shock wave intensity during the bubble collapse.

#### 3.1.2 Effect of salted water temperature on shock wave pressure of the bubble collapse

To analyze the variation of collapse shock wave intensity with increasing salted water temperature, this section would study the changes in pressure profile and pressure peak under different temperature conditions.

**Fig. 9:**
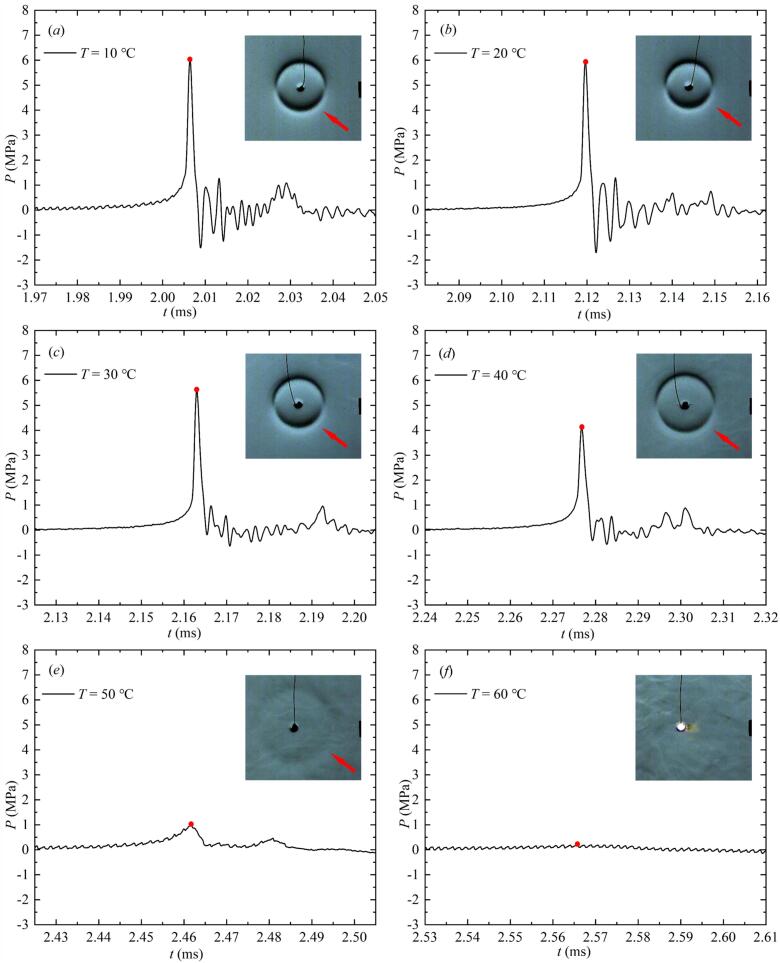
Effect of salted water temperature on the pressure profile of collapse shock wave from the bubble (the collapse shock wave front indicated by the red arrow in the high-speed photographic image, and the red solid dot indicated the pressure peak value of the collapse shock wave). (For interpretation of the references to colour in this figure legend, the reader is referred to the web version of this article.)

Fig. 9 showed the pressure profile of the collapse shock wave measured in salted water at different temperatures with a relative distance *γ*_1_ = 3. *γ*_1_ was the relative distance obtained by dividing the distance *L* between the pressure measuring point and the center of the bubble by the maximum radius *R*_max_ of the bubble. *P* was the impact pressure of the collapse shock wave measured at the measuring point. There are two reasons for choosing *γ*_1_ = 3 in this study: on the one hand, the measuring range of the sensor used in the experiment was limited, with a maximum range of 20 MPa. On the other hand, it could be seen from the literature [45] that the pressure peak of shock wave gradually decreased as the distance between the bubble and the sensor measuring point increased. If the sensor measuring point was close to the bubble center, the pressure peak of shock wave exceeded the measuring range of the sensor, and the sensor also affected characteristics of the bubble dynamics. Therefore, *γ*_1_ was set to 3 in this study.

As seen in Fig. 9 (*a*-*f*), with an increase in salted water temperature, the collapse shock wave pressure peak *P*_max_ gradually decreased. Correspondingly, the collapse shock wave front in the high-speed photography images weakened from a sharp state to a blurry state with increasing salted water temperature, and finally disappeared from the field of view. Notably, at a temperature of 70°C, the sensor barely measured the collapse shock wave pressure peak *P*_max_ in this study.

**Fig. 10:**
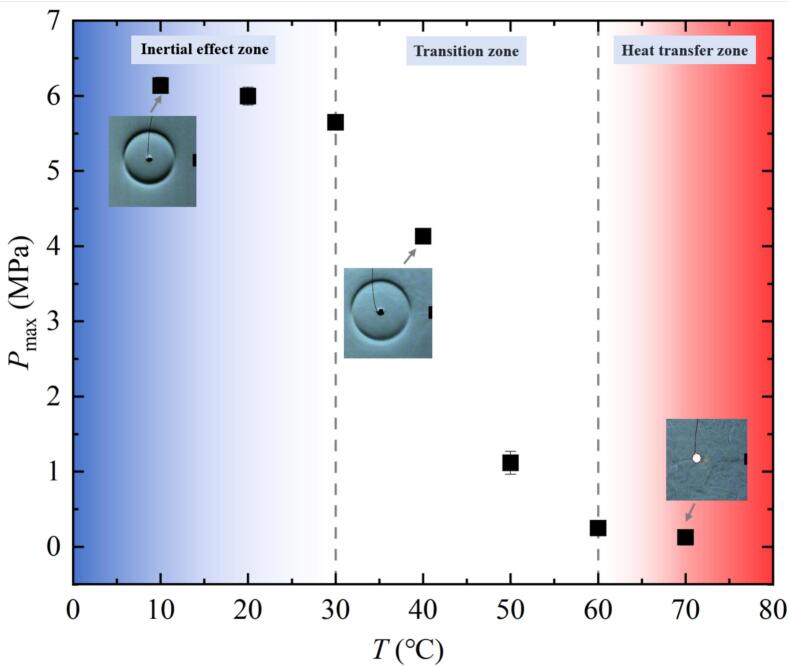
Effect of salted water temperature on the pressure peak of the collapse shock wave from the bubble (all data points in the figure had error bars).

The above findings showed that the pressure peak value of the collapse shock wave decreased gradually with increasing salted water temperature. Moreover, the higher the salted water temperature, the more obvious the decrease of *P*_max_ compared to the salted water at normal temperature. Next, this study would further analyze the variation of *P*_max_ with *T* in Fig. 10.

As can be seen from Fig. 10, *P*_max_ in the inertial effect zone gradually decreased with the increase of *T*. In the transition zone, *P*_max_ decreased sharply with the increase of *T*. *P*_max_ in the salted water at 70 ℃ was basically 0 MPa.

Based on the above experimental data, the effect of salted water temperature on the pressure of the collapse shock wave was mainly due to the reduced condensation rate as salted water temperature increased. This led to an increase in the minimum volume of the first collapse, more energy stored in the bubble, and less energy emitted as a shock wave into the surrounding salted water. As a result, the pressure peak of the collapse shock wave decreased with increasing salted water temperature.

#### 3.1.3 Effect of salted water temperature on energy configuration of the bubble

To evaluate the strength of the collapse shock wave, it was necessary to analyze the influence of salted water temperature increase on the collapse shock wave from the perspective of energy. The energy of shock wave could be obtained through the following formula [34]:

(4) $$E=\frac{4\pi L^{2}}{\rho_{0}c_{0}}\int P^{2}dt$$

In the formula, *L* is the distance from the center of the bubble to the measuring point, $\rho_{0}$ is the density of salted water, and $c_{0}$ is taken as the value corresponding to the water temperature [46]. The specific parameters of $\rho_{0}$ are shown in Fig. 2. The integration boundary of the collapse shock wave comprises the complete waveform. The waveform is the original one collected by the acquisition instrument, without filtering calculation.

This study further discussed the relationship between the energy *E*_s_ of the first collapse shock wave, the maximum mechanical energy *E*_max_ of the bubble, and the maximum mechanical energy *E*_r-max_ of the rebounding bubble. The calculation formula for mechanical energy is as follows [23,47]:

(5) $$E_{\max}=\frac{4\pi }{3}R_{\max}^{3}\left(p_{\infty }+\rho gh-p_{v}\right)$$

(6) $$E_{r-max}=\frac{4\pi }{3}R_{r-max}^{3}\left(p_{\infty }+\rho gh-p_{v}\right)$$

In the formula, $p_{\infty }$ is the ambient pressure, taken as 96 kPa; g is the acceleration of gravity, taken as 9.8 m/s^2^; h is the water depth between the salted water surface and the center of bubble, taken as 0.12 m, and $p_{v}$ is the saturated vapor pressure at the corresponding temperature.

**Fig. 11:**
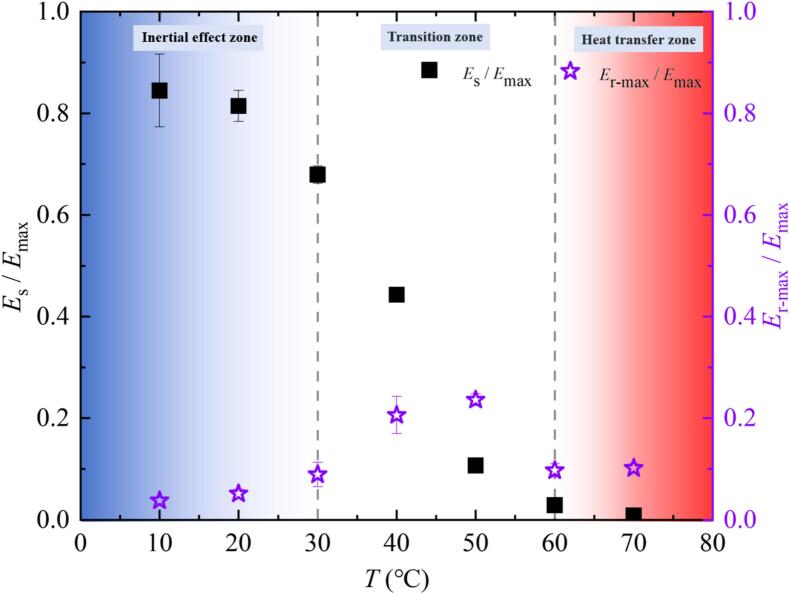
Effect of salted water temperature on energy configuration in the evolution of the bubble (all data points had error bars).

Based on the calculation of the above formula, Fig. 11 showed the relationship between the ratio (*E*_s_ / *E*_max_) and the ratio (*E*_r-max_ / *E*_max_) with salted water temperature *T*. In the black legend of Fig. 11, the ratio (*E*_s_ / *E*_max_) gradually decreased with the increase of *T* in the inertial effect zone. The ratio (*E*_s_ / *E*_max_) decreased significantly with the increase of *T* in the transition zone. In the heat transfer zone, when *T* was 70 ℃, the ratio (*E*_s_ / *E*_max_) was close to 0. In the purple legend, the ratio (*E*_r-max_ / *E*_max_) had a small increase with the increase of *T* in the inertial effect zone. the ratio (*E*_r-max_ / *E*_max_) firstly increased and then decreased with the increase of *T* in the transition zone. This was because *R*_r-max_ at 60 ℃ was smaller than *R*_r-max_ at 50 ℃. When *T* was 60 ℃ − 70 ℃, the ratio (*E*_r-max_ / *E*_max_) increased again with the increase of *T* in the heat transfer zone. In addition, in the experimental range of this study, when *T* was equal to or below 40 ℃, the ratio (*E*_s_ / *E*_max_) was greater than the ratio (*E*_r-max_ / *E*_max_). When *T* was equal to or greater than 50 ℃, the ratio (*E*_s_ / *E*_max_) was less than the ratio (*E*_r-max_ / *E*_max_).

The reason for the above changes was that the increase in salted water temperature led to an increase in its vaporization rate. The increase of vaporization rate would cause the increase of the maximum radius, the evolution period, the expansion time and the collapse time to increase. These changes further resulted in a decrease in the shock wave pressure peak and the ratio of the energy of the first collapse shock wave to the maximum mechanical energy of the bubble. And the increase in vaporization rate, the maximum rebound radius and the ratio of the maximum mechanical energy from the rebound bubble to the maximum mechanical energy of the bubble first increased, then decreased, and increased again. The trend of change in bubble energy loss due to phase transition was consistent with the literature [16,17]. The change in vaporization rate, surface tension, and viscosity changed the evolution, collapse characteristics, and energy configuration of the bubble in the free field, leading to similar changes in the microjet and shock wave of the bubble near the salted water surface.

### 3.2 Effect of salted water temperature on the bubble collapse behavior below the salted water surface

The microjet and the shock wave of the bubble are important indicators for analyzing the dynamics of the bubble below the salted water surface. Therefore, this section would conduct research on the effect of salted water temperature changes on the above two aspects.

#### 3.2.1 Effect of salted water temperature on the bubble microjet

**Fig. 12:**
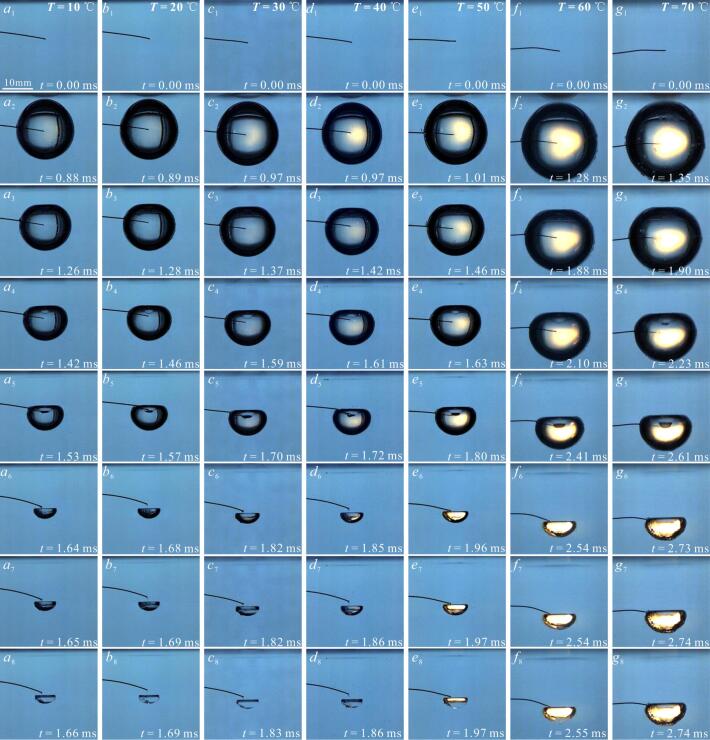
Development of the bubble microjet below the salted water surface at different salted water temperatures. The subscripts 1, 2, 3, 4, 5,6,7 and 8 for each operating condition represented the evolution process of the bubble. (shooting rate: 180,000 fps; exposure time: 3.95 µs; (*a*) *T* = 10 ℃; (*b*) *T* = 20 ℃; (*c*) *T* = 30 ℃; (*d*) *T* = 40 ℃; (*e*) *T* = 50 ℃; (*f*) *T* = 60 ℃; (*g*) *T* = 70 ℃).

Fig. 12 showed the high-speed photographic images of the bubble microjet evolution at different salted water temperatures when *γ*_2_ was about 1.2. Here, *γ*_2_ was the relative distance obtained by dividing the distance between the salted water surface and the center of the bubble by the maximum radius of the bubble. Within our experiments, *γ*_2_ = 1.2 was selected for two reasons: on the one hand, since the study primarily focused on the effect of liquid temperature on the microjet, *γ*_2_ was fixed as a constant value of 1.2. On the other hand, when *γ*_2_ = 1.2, the morphology of microjet is clear, making observation easier [29].

In Fig. 12 (*a*-*g*), the salted water temperature increased from 10 ℃ to 70 ℃. In Fig. 12 (*a*_3_-*g*_3_), the side of the bubble near the salted water surface was concave inward, generating the microjet [29,48,49]. In Fig. 12 (*a*_7_-*g*_7_), the microjet impacted the inner wall of the bubble far from the salted water surface. In Fig. 12 (*a*_8_-*d*_8_), the bubble shrank to the minimum volume.

**Fig. 13:**
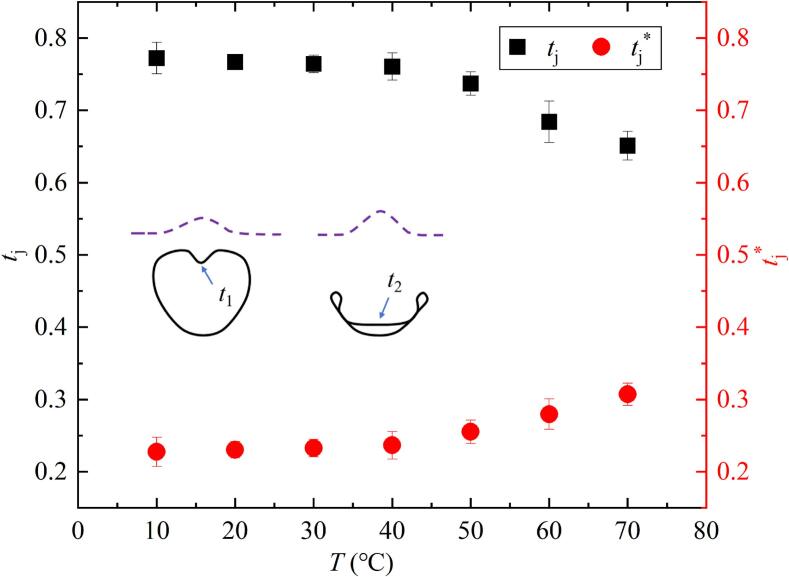
Effect of salted water temperature on the formation time (*t*_j_) and evolution time (*t*_j_*) of the bubble microjet (all data points had error bars).

Fig. 13 showed the effect of salted water temperature change on the dimensionless time *t*_j_ of the bubble generation to microjet appearance, and the dimensionless time *t*_j_* of the microjet evolution inside the bubble. The definitions of dimensionless time *t*_j_ and *t*_j_* are as follows:

(7) $$t_{j}\;=\;\frac{t_{1}}{t^{*}}$$

(8) $${t_{j}}^{*}\;=\;\frac{t_{2}-t_{1}}{t^{*}}$$

In the formula, *t*_1_ is the formation time from the bubble generation to the microjet appearance, and *t*_2_ was the evolution time from the bubble generation to the microjet impact on the inner wall of the bubble away from the salted water surface. The illustration in the figure roughly represented the evolution of microjet at selected time *t*_1_ and *t*_2_. t* was the first evolution period of the bubble.

In Fig. 13, it could be seen that *t*_j_ in the inertial effect zone and transition zone gradually decreased with the increase of *T*. *t*_j_* in the inertial effect zone and transition zone gradually increased with the increase of *T*, as shown in Fig. 13.

**Fig. 14:**
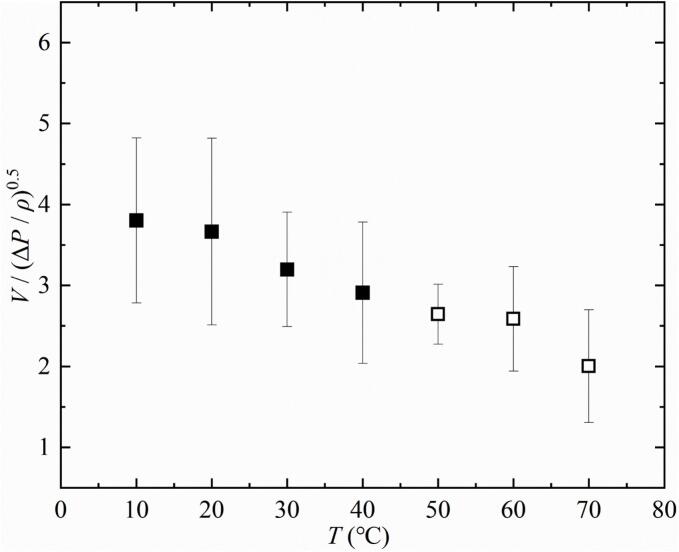
Effect of salted water temperature on dimensionless velocity of the microjet. (Under experimental conditions of 10°C − 40°C, the frame before the microjet impacted the inner wall of the bubble was used to calculate the microjet velocity *V*. When *T* is in the range of 50°C − 70°C, due to the intense luminescence inside the bubble, the calculation of the microjet velocity may not be sufficiently accurate. During the calculation of the microjet (50°C − 70°C), the last frame in which the microjet can be observed in high-speed photography is selected to calculate *V*. The characteristic velocity ${\left(\Delta P/\rho \right)}^{0.5}$ was used to nondimensionalize *V*.).

The microjet velocity is an important consideration factor in bubble dynamics research. Therefore, Fig. 14 showed the effect of salted water temperature increase on the bubble microjet velocity. From Fig. 14, it can be seen that as *T* increased (10 ℃ − 40 ℃), the nondimensional velocity $\left(V/{\left(\Delta P/\rho \right)}^{0.5}\right)$ of the bubble microjet gradually decreased. It should be noted that under the conditions of 50 ℃ − 70 ℃, the intense luminescence occurring during the bubble contraction phase had brought great difficulty to the extraction of microjet velocity. Therefore, when the temperature *T* was in the range of 50 ℃ − 70 ℃, the microjet velocity in the experiments of this study was not sufficiently accurate. However, judging from the overall trend of the microjet velocity varying with temperature, it gradually decreased as the temperature increased.

There may be two reasons for the above experimental results: on the one hand, due to the increase in salted water temperature, the saturated vapor pressure of the salted water increased [17,36], and the difference between the environmental pressure and the saturated vapor pressure gradually decreased [17], resulting in an increase in the evolution time of the bubble microjet. On the other hand, as the salted water temperature increased, the evolution time of the microjet induced by the same condition was longer, resulting in a longer effect of the salted water surface on the microjet, leading to a decrease in the dimensionless velocity of microjet. It was precisely because of the different effects of saturated vapor pressure and salted water surface on the drag of the bubble that the formation time, evolution time, and dimensionless velocity of the microjet undergo different changes. And this change also led to a change in the shock wave generated by the bubble evolution.

#### 3.2.2 Effect of salted water temperature on shock waves of the bubble

**Fig. 15:**
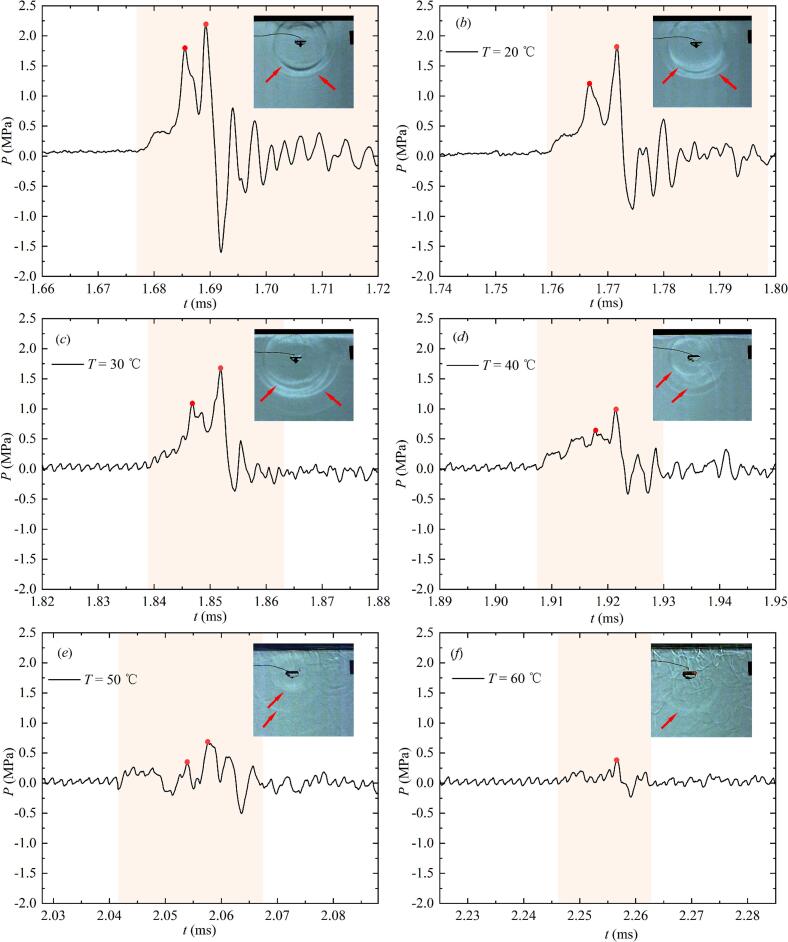
Effect of salted water temperature changes on the development of the shock waves (red arrows and red solid dots indicated wavefronts and pressure peaks of shock waves in the corresponding experiments; the light pink ribbon indicated the integral range for calculating the shock wave energy generated during the first collapse of the bubble). (For interpretation of the references to colour in this figure legend, the reader is referred to the web version of this article.)

Different from the bubble collapse behavior in the free field, shock waves generated when the bubble collapsed below the free liquid surface exhibited a multi-layered structural characteristic. Due to this multi-layered evolution pattern of shock waves, their energy had been dispersed in temporally and spatially. Therefore, Fig. 15 showed the pressure profiles of shock waves at different temperatures when *γ*_1_ was about 3 and *γ*_2_ was about 1.2. In Fig. 15 (*a*-*f*), the maximum pressure peak *P*_max_* of shock wave generated during the first collapse of the bubble gradually decreased with the increase of *T*. Due to limitations in measurement accuracy, it had become difficult to accurately measure the impact pressure process when the temperature exceeded 70 ℃.

**Fig. 16:**
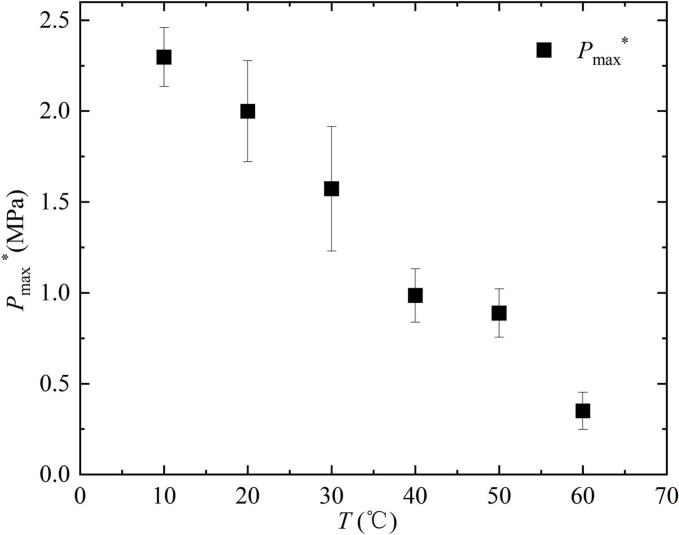
Effect of salted water temperature on the maximum pressure peak of the shock wave generated during the first collapse of the bubble.

According to the analysis in Fig. 15, salted water temperature changes could affect the pressure of the shock wave. The pressure peak is an important indicator for measuring the damage caused by the shock waves to the boundary. Therefore, Fig. 16 showed the variation of *P*_max_* with increasing salted water temperature. In Fig. 16, *P*_max_* decreased to varying degrees with the increase of *T*.

**Fig. 17:**
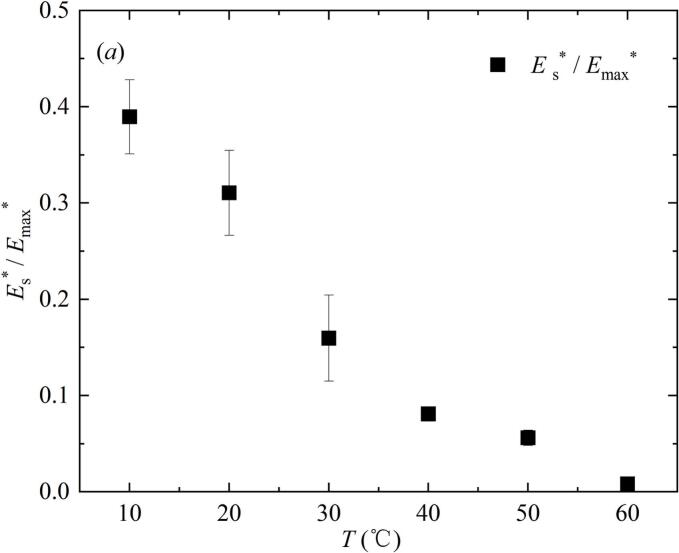
Effect of salted water temperature on the shock wave energy generated during the first collapse of the bubble.

Fig. 17 showed the effect of salted water temperature changes on the energy ratio *E*_s_* / *E*_max_*. *E*_s_* / *E*_max_* represented the ratio of shock wave energy *E*_s_* generated during the first collapse of the bubble to the maximum mechanical energy *E*_max_*of the bubble below the salted water surface. *E*_s_* was calculated using formula 4. *E*_max_* was calculated using formula 5. In Fig. 17, the value of *E*_s_* / *E*_max_* gradually decreased with the increase of *T*.

There are two fundamental reasons for the above experimental results: on the one hand, during the bubble contraction process, the fluid between the bubble and the salted water surface could not be replenished in time, which had an effect on the microjet [50]. On the other hand, when the salted water temperature increased, its condensation rate decreased, resulting in an increase in the minimum volume of the bubble. Furthermore, it led to a decrease in the pressure peak value and energy of the shock wave. The trend of change in bubble energy loss due to phase transition was consistent with the literature [16,17].

### 3.3 Effect of salted water temperature on secondary cavitation

Through analyzing evolution and collapse characteristics of the bubble in salted water at different temperatures, secondary cavitation can occur under certain conditions in both the free field and below the salted water surface.

#### 3.3.1 Secondary cavitation during the bubble expansion process in a free field

**Fig. 18:**
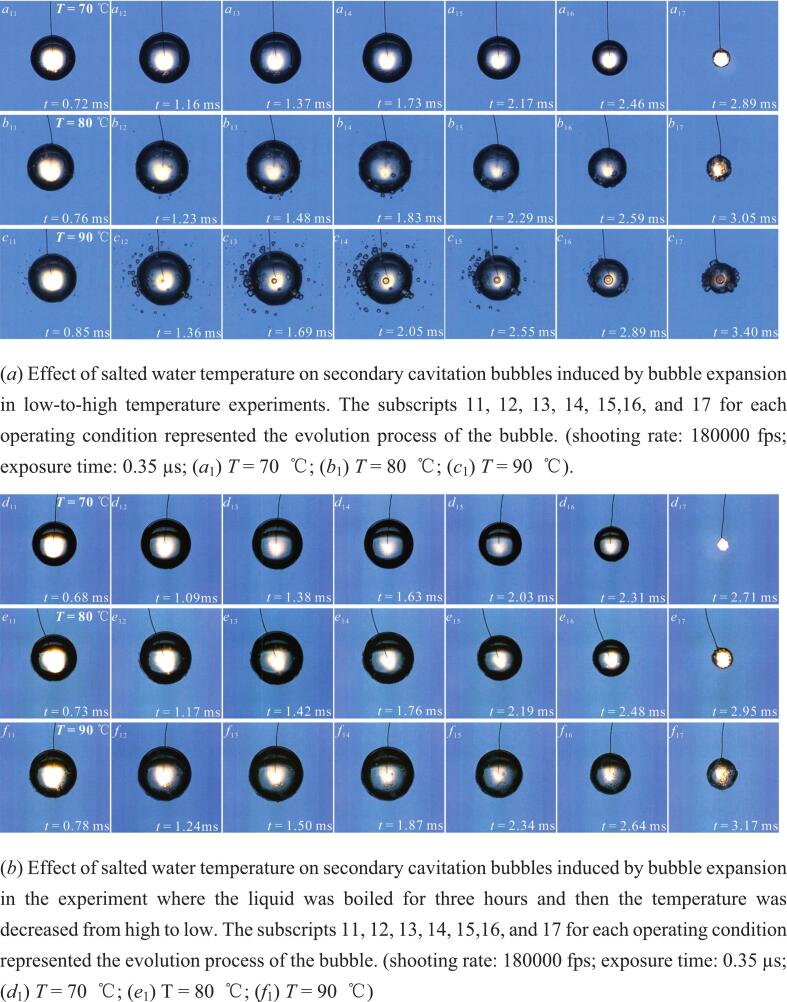

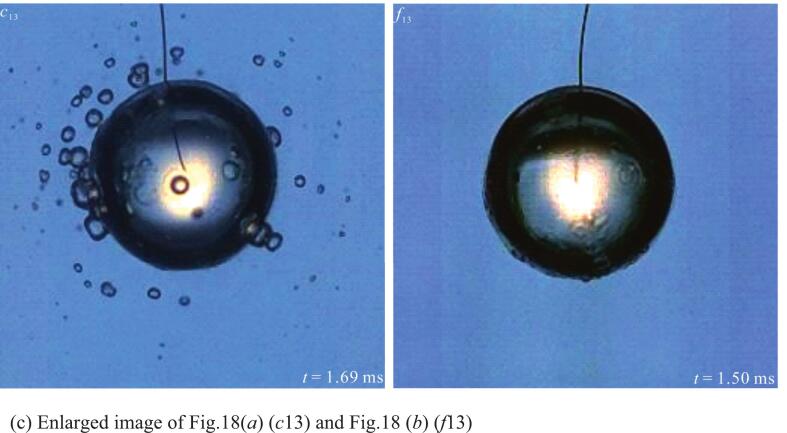
Secondary cavitation bubbles induced by the bubble expansion in salted water at different temperatures.

Fig. 18 (*a*) showed the secondary cavitation phenomenon during the bubble expansion process in the salted water temperature range of 70°C to 90°C. Fig. 18 (*a*) (*a*_13_-*c*_13_) showed the secondary cavitation phenomenon when the bubble expanded to its maximum volume. In Fig. 18 (*a*) (*a*_13_), a small number of secondary cavitation bubbles were generated on the bubble surface. In Fig. 18 (*a*) (*b*_13_-*c*_13_), secondary cavitation bubbles were generated on the bubble surface and in the surrounding liquid. And as the salted water temperature increased, the maximum volume and number of secondary cavitation bubbles also increased. Furthermore, the farther the distance from the bubble radially, the smaller the volume of secondary cavitation bubbles. The phenomenon of secondary cavitation occurring on the surface and surrounding areas of the bubble in high-temperature liquids was also observed in the literature [17]. Additionally, as shown in Fig. 18 (*a*) (*b*_16_-*c*_16_), during the bubble contraction process, the surrounding secondary cavitation bubbles also shrank.

The secondary cavitation phenomenon was related to the number of cavitation nuclei in the salted water. Therefore, in order to eliminate the influence of cavitation nuclei in the salted water as much as possible, this study carried out the reverse operation of the experiment in Fig. 18 (*a*). That is, the salted water was first boiled for three hours, and then experiments on the bubble were conducted in the order of salted water temperatures of 90°C, 80°C, and 70°C successively. The experimental results were shown in Fig. 18 (*b*). It could be observed that in Fig. 18 (*b*) (*d*_13_), when the bubble expanded to its maximum volume, only a very small number of tiny secondary cavitation bubbles were generated on its surface. In Fig. 18 (*b*) (*e*_13_-*f*_13_), when the bubble expanded to its maximum volume, obvious secondary cavitation bubbles were generated on the bubble surface, and no secondary cavitation bubbles were found in the surrounding salted water. Moreover, as the salted water temperature increased, both the maximum volume and the number of secondary cavitation bubbles on the bubble surface also increased. This was different from the secondary cavitation phenomenon during the bubble expansion process in Fig. 18 (*a*). However, it cannot be ruled out that the secondary cavitation phenomenon occurred on the surface of the bubble in the experiment of Fig. 18 (*b*). Therefore, in order to visually observe secondary cavitation bubbles around the bubble, this study enlarged Fig. 18 (*a*) (*c*_13_) and Fig. 18 (*b*) (*f*_13_), as shown in Fig. 18 (*c*).

**Fig. 19:**
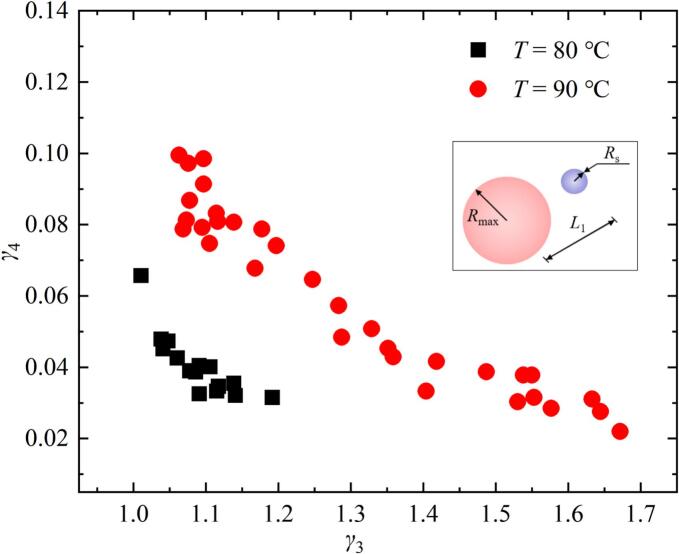
Variation of the relative size *γ*_4_ with the relative distance *γ*_3_ when the bubble expanded to its maximum volume at different temperatures.

From the above analysis, it can be observed that the intensity of the secondary cavitation varied under different temperature conditions. Therefore, this study further conducted a statistical analysis of secondary cavitation bubbles in the series of experiments Fig. 18 (*a*) (at salted water temperatures ranging from 80°C to 90°C), as shown in Fig. 19. Among them, *γ*_3_ was the distance *L*_1_ between the center of the secondary cavitation bubble and the center of the bubble divided by *R*_max_, and *γ*_4_ was the radius *R*_s_ of the secondary cavitation bubble divided by *R*_max_. In order to intuitively understand the definition of parameters, the illustration was drawn in Fig. 19. As can be seen from Fig. 19, at the same salted water temperature, *γ*_4_ decreased with the increase of *γ*_3_. Under conditions where *γ*_3_ was approximately the same, *γ*_4_ at 90 ℃ was greater than that at 80 ℃.

This study discovered that secondary cavitation can be induced during the bubble expansion process in high-temperature salted water experiments, which has not been observed in previous literature. There are two reasons for the above experimental results: on the one hand, during the bubble expansion, the surrounding salted water would move radially outward with the bubble, and the speed of salted water would decrease with the increase of the radial distance from bubble. As a result, the pressure of the salted water gradually increased with the increase of the radial distance from bubble, leading to a lower pressure at the bubble surface than the pressure of surrounding salted water. On the other hand, the saturated vapor pressure gradually increased as the salted water temperature increased [17,36]. Therefore, under the influence of both factors, secondary cavitation bubbles were more likely to occur near the bubble interface, and the number of secondary bubbles at higher temperatures would also increase. And the radius of the secondary bubble at the same temperature decreased with increasing radial distance from bubble. Under the same distance between the bubble and the secondary bubble, when the bubble expanded to its maximum radius, the radius of the secondary bubble at higher temperatures was greater than that at lower temperatures.

#### 3.3.2 Secondary cavitation during the bubble collapse process below the salted water surface

The above experimental results analyzed the secondary cavitation during expansion process of the bubble at higher temperatures in a free field. During the experiment, this study found that the secondary cavitation generated under high-temperature and low-temperature conditions were different when the bubble collapse below the salted water surface.

**Fig. 20:**
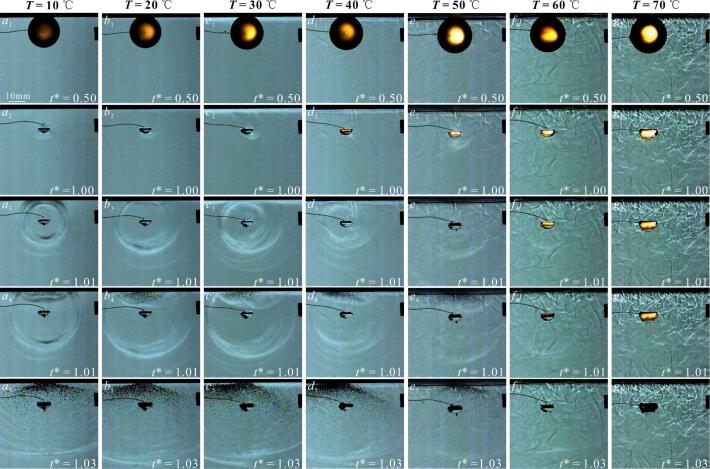
Secondary cavitation induced by shock wave of the bubble collapse below the salted water surface in salted water at different temperatures. The subscripts 1, 2, 3, 4, and 5 for each operating condition represented the evolution process of the bubble. (shooting rate: 180,000 fps; exposure time: 0.25 µs; (*a*) *T* = 10 ℃; (*b*) *T* = 20 ℃; (*c*) *T* = 30 ℃; (*d*) *T* = 40 ℃; (*e*) *T* = 50 ℃; (*f*) *T* = 60 ℃; (*g*) *T* = 70 ℃).

Fig. 20 showed secondary cavitation phenomenon induced by shock waves below the salted water surface. In Fig. 20 (*a*-*g*), the salted water temperatures were 10 ℃, 20 ℃, 30 ℃, 40 ℃, 50 ℃, 60 ℃ and 70 ℃, respectively. Fig. 20 (*a*_3_-*g*_3_) showed images of shock waves. Fig. 20 (*a*_5_-*g*_5_) showed the secondary cavitation generated below the salted water surface when shock waves impacted the salted water surface. In Fig. 20 (*a*_5_-*g*_5_), secondary cavitation was very obvious in salted water at 10°C − 40°C, stronger than the secondary cavitation at 50°C. Notably, when the salted water temperature increased to 60 ℃ and 70 ℃, there were basically no secondary cavitation bubbles below the salted water surface. The secondary cavitation below the salted water surface also appeared in the study by Supponen et al. [29]. The difference was that the salted water temperature studied by Supponen et al. [29] was normal temperature, and the bubble was very close to the salted water surface.

There are two reasons for the above phenomenon: on the one hand, the low-pressure generated by the reflection of shock waves after impacting the salted water surface [29], which led to the generation of secondary cavitation bubbles. On the other hand, as the salted water temperature increased, the peak value of the shock wave gradually decreased. After the shock wave impacted the salted water surface, different degrees of low-pressure were generated, leading to different amounts of secondary cavitation bubbles below the salted water surface.

## 4 Conclusion

In view of the lack of clear research on the effect of salted water temperature on the bubble evolution and collapse, this study used corona discharge method to induce cavitation bubbles, and in combination with a high-speed photography and high-frequency pressure testing system, systematically studied on the dynamic process and collapse behavior of the bubble in the salted water temperature range of 10 ℃ − 90 ℃. The following preliminary conclusions are obtained:

(1) The effect of the increase in salted water temperature on the dynamic process of the bubble showed three distinct zones. Through high-speed photography observations of the bubble, this study found that changes in salted water temperature, which affected the evaporation and condensation rates, led to a greater increase in the maximum bubble radius, first contraction minimum radius, expansion time, and collapse time in the 30 °C − 60 °C range compared to the 10 °C − 30 °C range, but a smaller increase compared to the 60 °C − 90 °C range. Further investigation revealed that the pressure peak of the shock wave and the ratio of shock wave energy to the maximum mechanical energy of the bubble decreased more significantly in the 30 °C − 60 °C range than in the 10 °C − 30 °C range. The decrease was much greater than in the 60 °C − 70 °C range.
(2) The increase in salted water temperature significantly affected the microjet velocity and shock wave intensity during the bubble collapse below the salted water surface. As the salted water temperature increased (10 ℃ − 40 ℃), the microjet velocity of the bubble gradually decreased. Moreover, as the salted water temperature increased, the maximum pressure peak of shock wave also showed a decreasing trend. At the same time, the energy of the shock wave also decreased as the salted water temperature increased.

Varying liquid temperatures altered the bubble dynamics, leading to changes in the asymmetric collapse characteristics induced by different boundaries. This had important theoretical significance for the efficient utilization of cavitation or the prevention of potential cavitation damage. Therefore, the new experimental findings in this study provide a new idea for realizing the technology of controlling cavitation intensity using temperature in practical cavitation scenarios.

## CRediT authorship contribution statement

**Guihua Fu:** Writing – original draft, Investigation, Data curation. **Jing Luo:** Writing – original draft, Methodology, Investigation, Formal analysis. **Weilin Xu:** Methodology, Conceptualization. **Jiguo Tang:** Methodology. **Hang Wang:** Methodology.

## Declaration of competing interest

The authors declare that they have no known competing financial interests or personal relationships that could have appeared to influence the work reported in this paper.
